# Sensitive detection of multiple islet autoantibodies in type 1 diabetes using small sample volumes by agglutination-PCR

**DOI:** 10.1371/journal.pone.0242049

**Published:** 2020-11-13

**Authors:** Felipe de Jesus Cortez, David Gebhart, Peter V. Robinson, David Seftel, Narges Pourmandi, Jordan Owyoung, Carolyn R. Bertozzi, Darrell M. Wilson, David M. Maahs, Bruce A. Buckingham, John R. Mills, Matthew M. Roforth, Sean J. Pittock, Andrew McKeon, Kara Page, Wendy A. Wolf, Srinath Sanda, Cate Speake, Carla J. Greenbaum, Cheng-ting Tsai

**Affiliations:** 1 Enable Biosciences Inc., South San Francisco, CA, United States of America; 2 Department of Chemistry, Stanford University, Stanford, CA, United States of America; 3 Stanford Diabetes Research Center, Stanford, CA, United States of America; 4 Division of Pediatric Endocrinology and Diabetes, Department of Pediatrics, Stanford University School of Medicine, Stanford, CA, United States of America; 5 Department of Laboratory Medicine/Pathology, Mayo Clinic, College of Medicine, Rochester, MN, United States of America; 6 Department of Neurology, Mayo Clinic, College of Medicine, Rochester, MN, United States of America; 7 T1D Exchange, Boston, MA, United States of America; 8 Diabetes Center, University of California, San Francisco, San Francisco, CA, United States of America; 9 Diabetes Clinical Research Program, Benaroya Research Institute, Seattle, WA, United States of America; Université Paris Descartes, FRANCE

## Abstract

Islet autoantibodies are predominantly measured by radioassay to facilitate risk assessment and diagnosis of type 1 diabetes. However, the reliance on radioactive components, large sample volumes and limited throughput renders radioassay testing costly and challenging. We developed a multiplex analysis platform based on antibody detection by agglutination-PCR (ADAP) for the sample-sparing measurement of GAD, IA-2 and insulin autoantibodies/antibodies in 1 μL serum. The assay was developed and validated in 7 distinct cohorts (n = 858) with the majority of the cohorts blinded prior to analysis. Measurements from the ADAP assay were compared to radioassay to determine correlation, concordance, agreement, clinical sensitivity and specificity. The average overall agreement between ADAP and radioassay was above 91%. The average clinical sensitivity and specificity were 96% and 97%. In the IASP 2018 workshop, ADAP achieved the highest sensitivity of all assays tested at 95% specificity (AS95) rating for GAD and IA-2 autoantibodies and top-tier performance for insulin autoantibodies. Furthermore, ADAP correctly identified 95% high-risk individuals with two or more autoantibodies by radioassay amongst 39 relatives of T1D patients tested. In conclusion, the new ADAP assay can reliably detect the three cardinal islet autoantibodies/antibodies in 1μL serum with high sensitivity. This novel assay may improve pediatric testing compliance and facilitate easier community-wide screening for islet autoantibodies.

## Introduction

Type 1 diabetes (T1D) is a chronic autoimmune disease, characterized by progressive destruction of pancreatic islet beta cells. T1D affects millions of Americans, with over 70,000 new cases arising each year. Over the past 20 years, the number of reported cases of T1D has doubled due to the global increase in incidence rate [1]. Symptomatic T1D often coincides with diabetic ketoacidosis (DKA), requiring emergency clinical intervention. The complications and chronic treatment of T1D impose major physical, emotional and financial burdens on people with diabetes and their families [2].

The presence of two or more islet autoantibodies defines the earliest stage of T1D [3]. Early identification of these Stage 1 individuals reduces the risk of DKA by permitting timely glycemic control, and facilitates enrollment in clinical trials during a critical window when patients still harbor islet cell function and are more likely to respond favorably to immunomodulatory intervention [4].

Furthermore, classical metrics based on body mass index (BMI) and age have reduced predictive power due to rising obesity among children and adolescents and an increased recognition of new-onset T1D in adults. Islet autoantibodies can thus aid in diabetes type differentiation and confirmation of autoimmune diabetes for optimal treatment [5].

Currently, radioassay remains the most common tool to detect islet autoantibodies. Radioassay preserves antigen conformations, permitting detection of cognate autoantibodies with high sensitivity. However, a solution-phase autoantibody assay that does not require radioactive components, decreases the blood volume consumption and increases assay throughput (e.g. shorter time to result) could greatly improve testing capacity and compliance, especially in pediatric populations.

Alternative methods such as ELISAs have been developed to address some of the limitations of radioassay; however, they do not detect insulin autoantibodies, one of the earliest biomarkers for type 1 diabetes, and still consume 20–50 μL of serum per autoantibody [6–8]. Other multiplex approaches such as nanoparticle enhanced immunoassays and electrochemical luminescence (ECL) assays require specialized instrumentation for assay readout, sample pre-treatment or longer time-to-result (>16 hr) [5, 9–13]. Thus, detection of islet autoantibodies for disease diagnosis and risk screening can benefit substantially from a sample-sparing, non-radioactive, multiplexed, highly sensitive/specific assay that is easily deployable in routine clinical laboratories.

Antibody detection by agglutination-PCR (ADAP) represents a new platform to measure multiple antibodies/autoantibodies with high sensitivity [14, 15]. The ADAP assay leverages the multivalency of antibodies/autoantibodies to aggregate antigen-DNA conjugates into close proximity, positioning them for ligation upon introduction of a “bridge oligo” which is complementary to both DNA strands. In this way, the PCR-amplifiable DNA is only formed upon binding of autoantibodies to their antigens, and PCR readout directly reflects this binding event. The agglutination and ligation steps of ADAP therefore help to circumvent issues other PCR-based immunoassays have faced by requiring binding events to generate a “turn-on” signal, rather than having DNA attached directly to the antibody as in immuno-PCR. Together, these innovative aspects of ADAP increase the fidelity of this PCR-based assay by dramatically reducing background and improving signal. Additionally, this simple assay does not require any washing and centrifugation protocols to remove unbound secondary reporters.

Herein, we report the development and large-scale testing of an improved ADAP method to measure multiple islet autoantibodies in 1 μL serum samples that meets the requirements for T1D. The assay was tested in multiple blinded study cohorts, including subjects with T1D, their relatives and individuals with other diseases (S1 Table). The sample-sparing assay helps promote testing compliance, and allows researchers to preserve precious samples for a wide range analyses such as transcriptome, genome, metabolism and cellular immunity to facilitate immune signature identification [16, 17].

## Results

### The ADAP assay is capable of measuring antibodies against islet antigens

**Fig 1:**
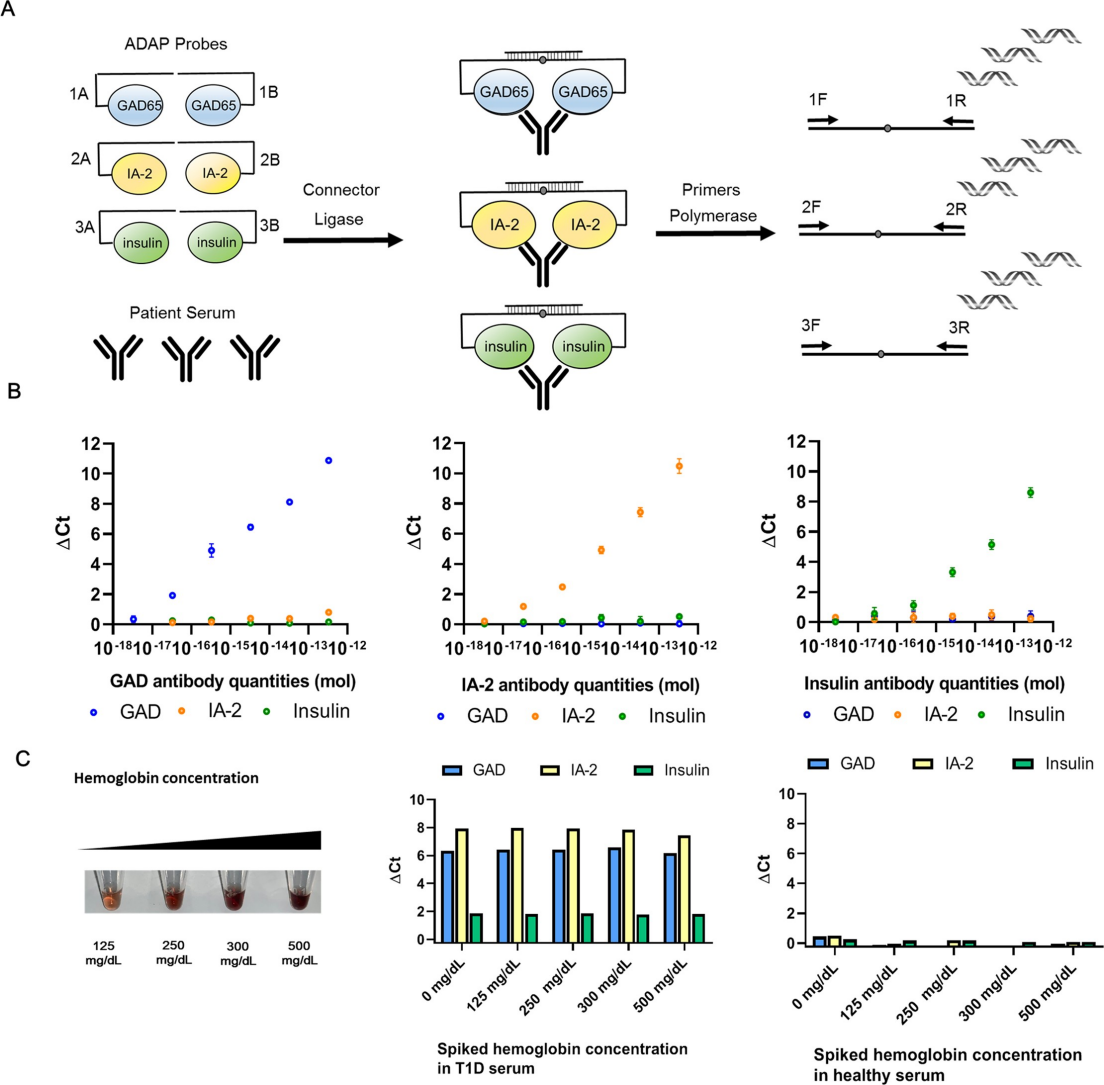
Schematic of sample-sparing ADAP assay for detection of multiple islet autoantibodies and the analytical characterization. **(A)** The workflow of ADAP is consisted of three steps. First, 1μL of serum is incubated with islet antigen conjugate probes harboring distinct DNA barcode pairs for 30 min. The multivalency of target autoantibodies agglutinates the cognate antigen-DNA conjugate pairs into close proximity. Secondly, the addition of a ligase and a bridge oligonucleotide reunites the two-separated barcode pairs into a full length amplicon. Finally, the ligated product is PCR amplified and then quantified with distinct primer pairs in the RT-PCR. Since each antigen-DNA conjugate only has one primer binding site and thus not PCR amplifiable on its own, no washing or centrifugation step is needed to remove unreacted probes. (**B**) The multiplex ADAP assay detected cognate antibodies without cross-reactivity. From left to right, antibodies from immunized animals against GAD, IA-2 and insulin were serially diluted and assayed by ADAP. The x-axis displays the quantities of the antibodies in the sample. The y-axis is ΔCt calculated by the difference of Ct value between the sample and a blank (S7 Fig). Signals for GAD, IA-2 and insulin antibodies are color coded in blue, orange and green respectively. Error bars represent standard deviation from triplicate, but for many data points are too small to be visualized. (**C**) Tolerance of ADAP for common blood contaminant was investigated by spiking hemoglobin at various concentration in T1D and healthy serum. No interference is observed up to 500 mg/dL of hemoglobin.

To develop an ADAP assay to measure islet autoantibodies with high accuracy, we needed to produce high quality antigen-DNA conjugate probes (Fig 1A). To that end, recombinant full length glutamic decarboxylase (GAD), full length insulin and cytoplasmic part of islet antigen-2 proteins (IA-2, amino acid 604–979) that contained major epitopes for islet autoantibodies were obtained. The first challenge we encountered was the susceptibility of proteins to aggregation and potential denaturation under the standard DNA installation process by SMCC (succinimidyl 4-(N-maleimidomethyl)cyclohexane-1-carboxylate) chemistry, likely due to the fact that these autoantigens were highly conformational and contained several hydrophobic regions. This issue was effectively mitigated by reducing the molar ratio of SMCC over proteins, and high quality two DNA per protein conjugates were obtained as affirmed by UV-VIS spectroscopy and SDS-PAGE characterization (S1 Fig).

The second challenge that remained to be addressed was whether the installation of DNA on these autoantigens had blocked epitopes critical for antibody binding. To evaluate this impact, we obtained polyclonal antibodies against GAD, IA-2 and insulin from immunized animals. We chose polyclonal antibodies as opposed to monoclonals, because they contain more diverse epitope binding specificities, thus better mimicking real-world heterogeneity and complexity. Much to our satisfaction, we observed strong concentration dependent curves for all three antibodies (Fig 1B). The fact that each conjugate only generated signals toward cognate antibodies also demonstrated the strong specificity of the multiplex detection system.

Nevertheless, animal-derived antibodies may be different in nature to autoantibodies from actual patients. Thus, we further characterized the ADAP T1D assay with a panel of 200 control and 120 established T1D patients (S2 Fig). Reassuringly, the signal distribution between patients versus controls reached statistical significance (p<0.05). We then set the assay cutoffs to 99 percentile of the controls, and used several study cohorts described below to independently evaluate the performance of the system. These data together demonstrated the first evidence that ADAP assay could detect multiple disease relevant antibodies while only consuming 1μL of samples.

Finally, clinical specimens may be hemolyzed or lipemic, posing a challenge for antibody detection systems. As PCR-based assays were known to be sensitive to contaminant inhibitors in the samples, it was of great interest to characterize the ADAP method under these challenging conditions. Therefore, hemoglobin, lipids and bilirubin were spiked into T1D and normal control serum samples at various concentrations. Satisfactorily, GAD, IA-2 and insulin signals in the T1D specimens remained largely unchanged under all conditions, and the normal serum samples showed consistently low signals close to the zero baseline (Fig 1C and S3 Fig). Notably, spiked samples with highest hemoglobin, lipids and bilirubin concentration appeared more perturbed beyond typical hemolysis or lipemic specimens. The ability of ADAP to withstand such large quantities of interfering substances stems from the ligation step, where the 3μL of sample/conjugate mixture was expanded into 117μL of ligation solution. This volumetric dilution greatly reduced the impact of potential inhibitors on downstream PCR steps. These data further strengthen the applicability of the ADAP T1D methods for difficult sample types.

Taken together, a careful and stepwise development process has led to a sample-sparing multiplex assay that can detect antibodies related to T1D even in the presence of common blood contaminants.

### Sample-sparing ADAP T1D assay performance in an assay validation panel

Beyond the strong analytical foundation above, the clinical performance of ADAP remained to be evaluated. To this end, we obtained archived serum samples from type 1 diabetes patients (n = 30) and healthy controls (n = 39) from the Benaroya Research Institute (cohort 1). These samples had been analyzed by gold standard radioassays, offering a unique opportunity to benchmark ADAP performance. For this cohort, those performing and analyzing the ADAP assay were not masked to disease status or radioassay results.

**Fig 2:**
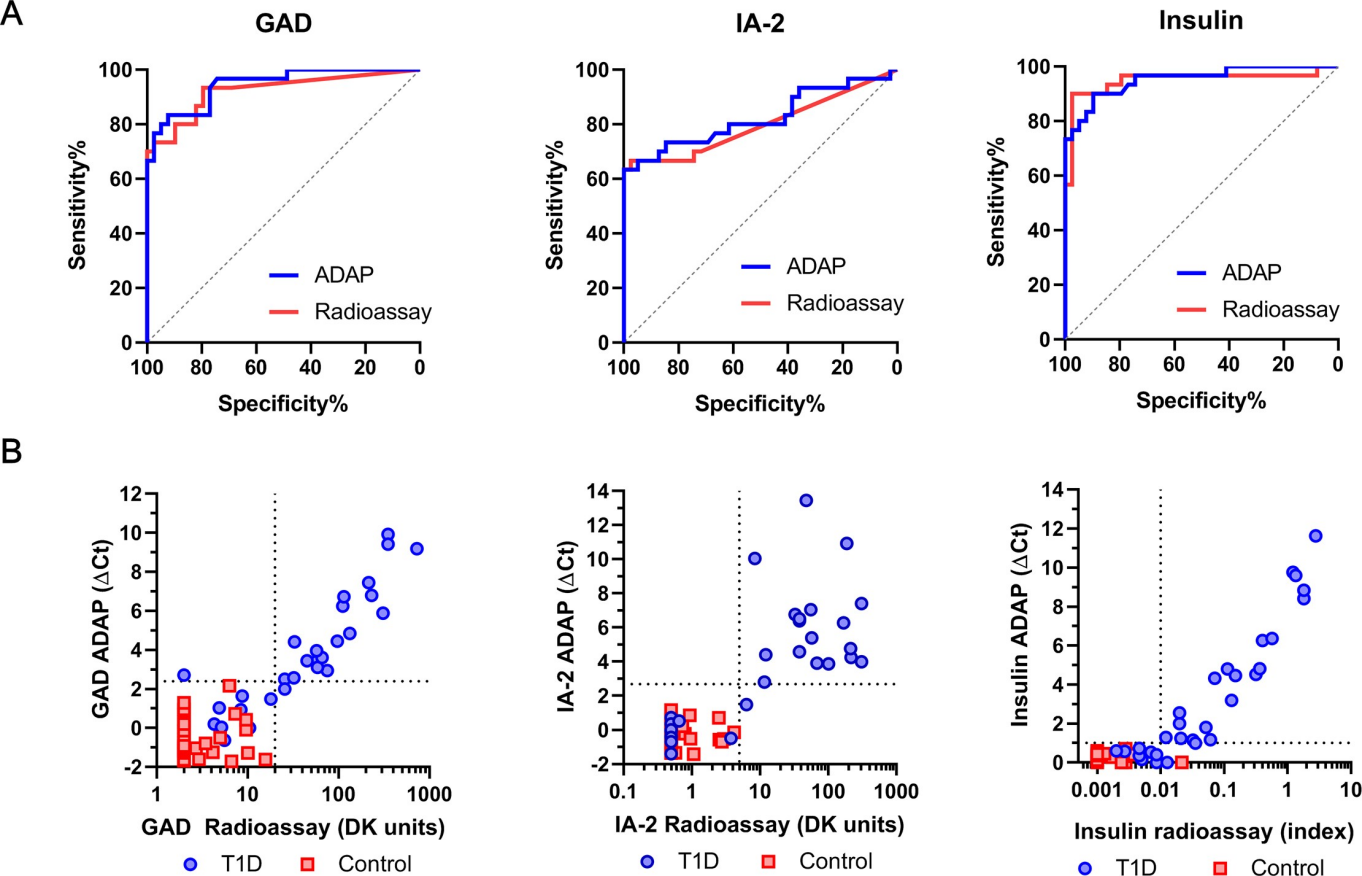
Clinical performance of ADAP assay in an evaluation cohort of T1D and healthy control. (A) The ROC curves of ADAP showed AUC of 0.94 (95%CI: 0.89–0.99), 0.82 (95%CI: 0.71–0.93) and 0.95 (95%CI: 0.90–1.00) for GAD, IA-2 and insulin antibodies/autoantibodies respectively. The samples were also analyzed by radioassay and showed corresponding AUC of 0.92 (95%CI: 0.85–0.99), 0.80 (95%CI: 0.68–0.92) and 0.95 (95%CI: 0.88–1.00). (B) Comparison plots of ADAP and radioassay signals. The x-axis displays radioassay signals in logarithm scales. The y-axis shows ADAP signal in ΔCt. The use of logarithm was necessary as ΔCt is a logarithmic parameter. (For instance, consider a sample of ΔCt value 2 and another sample of ΔCt of 4, their amplicon quantities differ by 4 fold (24/22) rather than 2 fold). The horizontal and the vertical dash lines denote ADAP and radioassay cutoff thresholds respectively. T1D sample data is shown in blue circle, whereas health serum signal is shown in red square. A total of 30 T1D and 39 control was analyzed without blinding.

We first compared autoantibody signals measured by the multiplex ADAP T1D assay and radioassay (Fig 2B). The Cohen’s kappa test showed that the κ coefficient were 0.89, 0.92 and 0.86 for GAD, IA2 and insulin autoantibodies/antibodies, indicating high concordance. Similarly, the overall agreement ranged from 93%-97% for three autoantibodies/antibodies. Beyond from concordance, the correlation analysis revealed Pearson’s R coefficients of 0.82, 0.69 and 0.88 for GAD, IA-2 and insulin autoantibodies, respectively.

The clinical sensitivity and specificity were 93% and 100% for ADAP compared to 93% and 98% for radioassay. In addition, the area under the curve (AUC) of the receiver operating characteristic (ROC) curves were 0.94 (95%CI: 0.89–0.99), 0.82 (95%CI: 0.71–0.93) and 0.95 (95%CI: 0.90–1.00) for GAD, IA2 and insulin autoantibodies/antibodies respectively (Fig 2A). Notably, the ROC curves of ADAP largely overlapped with those from radioassays (p>0.05), implying similar accuracy. Thus, these the results demonstrated that the ADAP assay could detect multiple islet autoantibodies/antibodies in 1μL serum with results consistent and concordant with radioassays.

### Sample-sparing ADAP T1D assay performance in the international Islet Autoantibody Standardization Program (IASP) 2018

With the solid in-house validation results, we next sought to rigorously evaluate the sample-sparing ADAP assay in a blinded manner by participating in the Islet Autoantibody Standardization Program (IASP) [18]. Firstly, the IASP study featured participants from over 30 laboratories from 17 countries. Secondly, aside from gold standard radioassays, other assays including ELISA, ECL, microarray, luciferase-based immunoprecitation system (LIPS) and Luminex microbeads also participated. Thirdly, each laboratory received a panel of coded serum from 43 new onset T1D, 7 first degree relatives of T1D with multiple islet autoantibodies and 90 normal controls. Notably, the new onset T1D samples were collected within 14 days of treatment so that any observed insulin autoantibodies were not simply antibodies against therapeutically-administered insulin. Finally, since the sample panel sent to each laboratory was coded differently, cross-comparing results between labs was not possible without the key from the committee. The IASP study was therefore an ideal approach to understand the capability of ADAP to detect disease relevant autoantibodies with no or minimal bias.

The unmasked results from the IASP committee revealed class-leading performance for ADAP (Table 1). The sensitivity of the ADAP assay at 95% specificity (termed AS95) were 88%, 74% and 66% for GAD, IA-2 and insulin autoantibodies respectively. When compared to all participating assays by AS95, the sample-sparing ADAP assay had the highest reported sensitivity for GAD and IA2 autoantibodies. For insulin autoantibodies, the ADAP AS95 value was 66% while the highest reported AS95 value was 68%. The IASP results definitively affirmed the high performance of the sample-sparing ADAP type 1 diabetes assay in detecting islet autoantibodies in 1μL serum samples.

**Table 1:** The ADAP assay performance in IASP 2018 study.

Islet cell Autoantibody Standardization Program (IASP) 2018			
		AS95 (Sensitivity at 95% specificity)	
	GAD Ab	IA-2 Ab	Insulin Ab
ADAP	88%	74%	66%
Reported Maximum	88%	74%	68%

The sensitivity at 95% specificity of ADAP is shown at the top, whereas the bottom values shows highest reported sensitivity among all participating laboratories worldwide using various testing methods. A total of 43 T1D, 7 high-risk relatives of T1D and 90 controls were analyzed with blinding.

### Evaluation of sample-sparing ADAP T1D assay using blinded T1D and T2D samples

Previous cohorts were based on T1D and normal controls. Generally a healthy individual with low risk factors rarely receives testing for islet autoantibodies. Instead, measurement was more likely to occur when a person developed a metabolic condition such as glucose intolerance.

**Fig 3:**
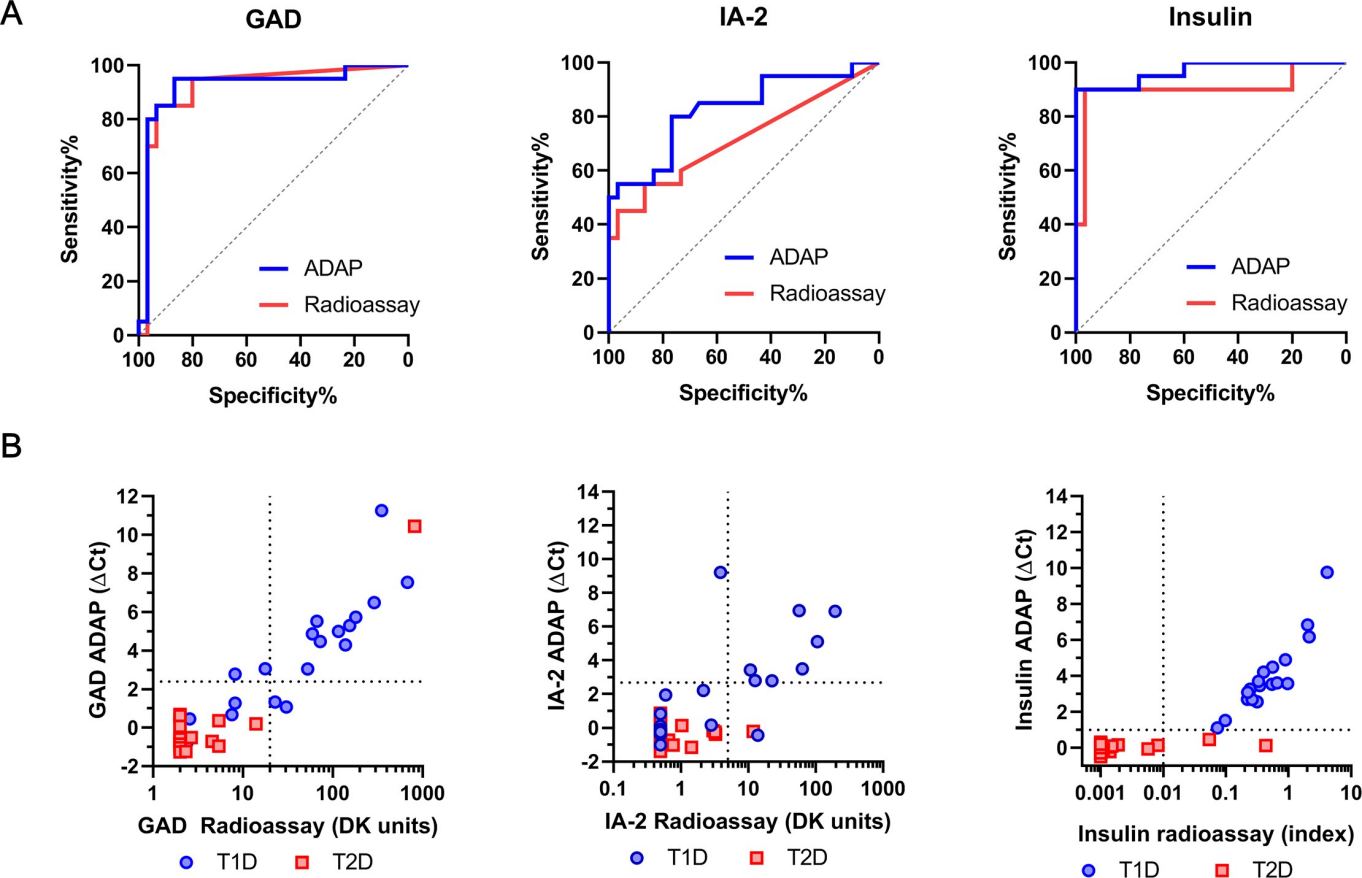
Validation of ADAP performance in a cohort of T1D and T2D samples. (A) The ROC curves of ADAP showed AUC of 0.92 (95%CI: 0.83–1.00), 0.83 (95%CI: 0.70–0.95) and 0.97 (95%CI: 0.92–1.00) for GAD, IA-2 and insulin antibodies/autoantibodies respectively. The samples were also analyzed by radioassay and showed corresponding AUC of 0.91 (95%CI: 0.82–1.00), 0.72 (95%CI: 0.56–0.87) and 0.90 (95%CI: 0.79–1.00). (B) Comparison plots of ADAP and radioassay signals. The x-axis displays radioassay signals in logarithm scales. The y-axis shows ADAP signal in ΔCt. The horizontal and the vertical dash lines denote ADAP and radioassay cutoff thresholds respectively. T1D sample data is shown in blue circle, whereas health serum signal is shown in red square. This cohort included 20 T1D and 30 T2D, and was analyzed with blinding.

To characterize the ADAP performance in such setting, we obtained a panel of masked serum samples derived from 20 individuals with T1D and 30 with T2D from the Benaroya Research Institute (cohort 3). The results were sent out to BRI investigators who then unmasked the samples and provided disease status and radioassay signals for analysis (Fig 3A).

For GAD, IA-2 and insulin autoantibodies/antibodies, the AUC of ROC were 0.92 (95%CI: 0.83–1.00), 0.83 (95%CI: 0.70–0.95) and 0.97 (95%CI: 0.92–1.00) (Fig 3B), the Cohen’s κ coefficient of concordance with radioassays were 0.80, 0.73, 0.87, the overall agreement with radioassays were 92%, 92% and 92%, and the Pearson’s correlation coefficient R were 0.86, 0.71 and 0.79, respectively. Subjects with T1D from this cohort were all positive for at least one autoantibody by ADAP and radioassays. On the other hand, 97% and 87% of samples from T2D patients were negative for all three autoantibodies by ADAP and radioassay respectively. The results indicated that ADAP had at least as good performance as standard assays in this clinically relevant population.

### Validation of samples-sparing ADAP T1D assay with control samples from patients without diabetes

Apart from testing in patients with metabolic conditions, we wished to further evaluate the specificity of ADAP in a challenging cohort comprised of subjects with active inflammation. Therefore, we obtained 60 coded serum samples from the Mayo Clinic (Cohort 5). This panel was consisted of 20 T1D serum samples, 20 normal controls, 14 samples from patients with hyperglobulinemia and 6 with systemic lupus erythematosus (SLE). Sera from patients with hyperglobulinemia exhibit elevated immunoglobulin levels due to acute or chronic infection and posed a challenge for traditional immunoassay analysis [19], while sera of SLE patients often possess poly-reactive autoantibodies arising from multisystem inflammation [20, 21].

The ADAP assay correctly identified all 20 of the T1D diagnosis samples and 37 of 40 control samples (Table 2). Only three hyperglobulinemia serum samples showed single positive signals for GAD autoantibodies close to the cutoffs. Their ADAP signals over cutoff ratios (S/C) were 1.35, 1.92 and 2.68. Since GAD autoantibodies are commonly associated with neurological disorders such as Stiff-person syndrome [21], it is possible that hyperglobulinemia patients could harbor such autoantibodies as well. To our knowledge, the frequency of GAD autoantibodies in the setting of hyperglobulinemia has not been rigorously characterized and reported in the literature.

**Table 2:** Analysis of a challenging sample cohort with ADAP assay.

Suspected T1D			Control			SLE			HG		
GAD Ab											
	Rad +	Rad -		Rad +	Rad -		Rad +	Rad -		Rad +	Rad -
ADAP+	7	2		0	0		0	0		0	3*
ADAP-	0	11		0	20		0	6		0	11
IA-2 Ab											
	Rad +	Rad -		Rad +	Rad -		Rad +	Rad -		Rad +	Rad -
ADAP+	6	1		0	0		0	0		0	0
ADAP-	0	13		0	20		0	6		0	14
INS Ab											
	Rad +	Rad -		Rad +	Rad -		Rad +	Rad -		Rad +	Rad -
ADAP+	9	4		0	0		0	0		0	0
ADAP-	0	7		0	20		0	6		0	14

A total of 20 T1D, 20 controls, 6 systemic lupus erythematosus (SLE) and 14 hyperglobulinemia (HG) serum samples were included in this cohort to determine the off-target propensity of ADAP in a population with autoimmune and/or inflammatory disorders. Only 3 HG patients showed GAD autoantibodies by ADAP (*The HG samples have not been tested by radioassays, and were presumed to be radioassay negative). This cohort was analyzed with blinding. Radioassay was abbreviated as Rad.

To further compare the correlation and concordance of ADAP to radioassay performed at the Mayo Clinic, we received another 80 coded serum samples (Cohort 6). These samples were fully blinded, and originally submitted for testing at the Mayo Clinic to establish T1D diagnosis. For GAD, IA2 and insulin autoantibodies/antibodies, the concordance coefficients κ with radioassay were 0.75, 0.83, 0.82, while overall agreement was 88%, 96% and 91%, and Pearson’s correlation coefficients (R) were 0.93, 0.93 and 0.93 respectively (S4 Fig).

Taken together, the results showed that the sample-sparing ADAP T1D assay identifies autoantibodies sensitively with high specificity even within a complex patient population with a variety of potentially confounding diseases.

### Evaluation of sample-sparing ADAP T1D assay in an at-risk cohort

In addition to confirmation of T1D diagnosis, islet autoantibodies have important utility for identification of high-risk individuals [3, 4]. Subjects with two or more islet autoantibodies are categorized as high-risk, because they have a lifetime risk of developing T1D approaching 100% [3]. Our previous validation efforts focused on analyzing samples from T1D patient post-diagnosis. Since autoantibody titers and epitope patterns may change as the disease progresses [22], we then further investigated ADAP performance in sera from pre-symptomatic at-risk subjects.

We obtained 39 coded serum samples from at-risk relatives of T1D patients from Stanford Medical School (Cohort 7). These serum samples were analyzed by radioassay in a reference laboratory. The sample’s autoantibody patterns were masked at the time of ADAP analysis.

**Fig 4:**
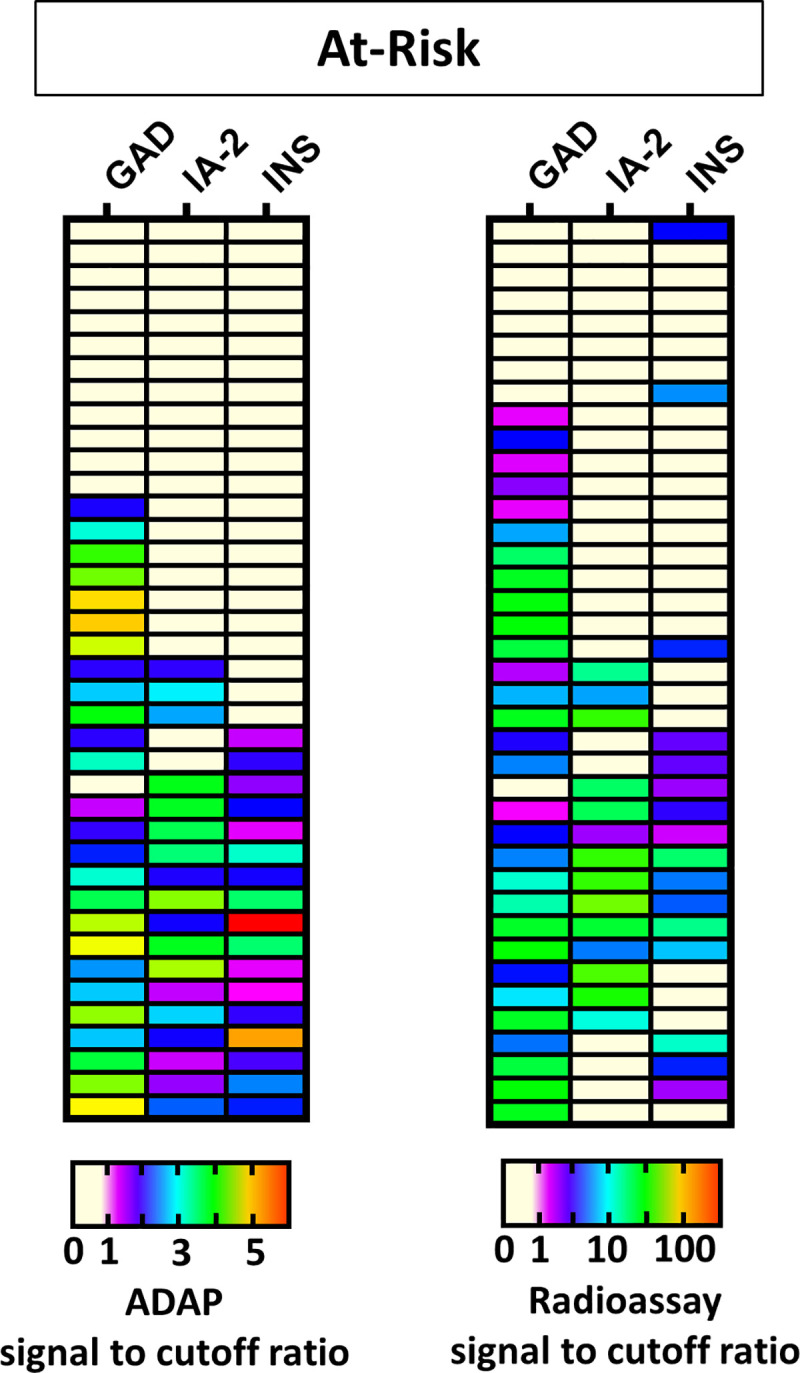
Heatmap of islet autoantibody patterns in at-risk T1D. Serum samples (n = 39) were analyzed by ADAP (left) and radioassay (right). ADAP and radioassay data was divided by corresponding cutoffs and plotted according to the color key at the bottom. Patients positive for two or more autoantibodies are at high-risk of progression to clinical onset of T1D. Radioassays identified 20 high-risk individuals, and 19 of those were also positive by ADAP, indicating ADAP’s ability for risk identification using 1μL of serum sample. This cohort was analyzed with blinding.

The result in the heatmap (Fig 4) showed that ADAP successfully detected 95% of high risk relatives with two or more autoantibodies by radioassays. Notably, among these radioassay double positive patients, 6 were triple positive by ADAP.

Intriguingly, for relatives initially classified as having a lower risk of progression (i.e. being single autoantibody positive by radioassay), ADAP reclassified 6 of these individuals to autoantibody negative. Two of them were low insulin autoantibody positive and four of them were low GAD autoantibody positive by radioassays. Future analysis of longitudinal samples, such as those from DAISY, TEDDY and TrialNet, are required to further investigate whether this reclassification can help improve the predictive accuracy of disease progressor versus non-progressor.

We also further obtained coded sera from 18 new onset T1D, 32 established T1D and 50 controls from Stanford School of Medicine. Consistent with previous results, ADAP correctly identified control patients to be negative for islet autoantibodies, and detected the majority of radioassay positive T1D samples with a tendency to identify additional positivity (S5 Fig).

Combined, the data demonstrated that ADAP can faithfully identify high-risk patients using just 1 μL of serum—a tiny volume that may greatly enhance screening compliance in pediatrics.

### Cross-cohort analysis of results between sample-sparing ADAP and radioassay

To summarize the results from the aforementioned cohorts, we first analyzed the accuracy of ADAP. The clinical sensitivity of ADAP ranged from 93%-100% and the specificity from 93%-100% (S2 Table). Then we pooled the data from those cohorts tested by radioassay at the same reference laboratory (cohort 1, 3 and 6), and showed that AUC of ROC were 0.91 (CI95%: 0.87–0.95), 0.82 (CI95%: 0.76–0.88) and 0.95 (CI95%: 0.92–0.98) for GAD, IA-2 and insulin autoantibodies/antibodies by ADAP, while the AUC of ROC were 0.91 (CI95%: 0.86–0.95), 0.83 (CI95%: 0.77–0.89) and 0.96 (CI95%: 0.93–0.99) for the same autoantibodies/antibodies by radioassays (S6 Fig). They largely overlapped and were statistically indistinguishable.

In addition, we also analyzed autoantibody patterns identified by ADAP and radioassays based on T1D status (Table 3). This analysis excluded IASP cohort 2 since the radioassay data of individual samples from participating laboratories were not public available. For new onset and established patients, ADAP and radioassays pattern were largely consistent, and ADAP correctly identified an additional 4–5% of patients considered autoantibody negative by radioassay. Importantly, 95% of samples from high-risk relatives were concordantly positive for two or more autoantibodies by ADAP and radioassay.

**Table 3:** Number of people found autoantibody positivity by the multiplex ADAP and radioassay.

At-risk		ADAP				
		3 Ab	2 Ab	1 Ab	0 Ab	
Radioassay	3 Ab	6	-	-	-	6
	2 Ab	7	6	1	-	14
	1 Ab	1	-	6	6	13
	0 Ab	-	-	-	6	6
		14	6	7	12	
New onsets		ADAP				
		3 Ab	2 Ab	1 Ab	0 Ab	
Radioassay	3 Ab	11	-	-	-	11
	2 Ab	2	2	-	-	4
	1 Ab	-	-	-	-	0
	0 Ab	-	1	-	2	3
		13	3	0	2	
Established		ADAP				
		3 Ab	2 Ab	1 Ab	0 Ab	
Radioassay	3 Ab	64	15	1	-	80
	2 Ab	25	89	13	-	127
	1 Ab	3	22	41	-	66
	0 Ab	-	1	7	21	29
		92	127	62	21	

Data from all of the assay validation cohort were pooled, except for IASP 2018 (cohort 2) because radioassay data for individual samples was not public available. There were a total of 39 at-risk relatives of T1D, 18 new onset T1D and 182 established T1D included in the analysis.

In summary, the data demonstrated that the sample-sparing ADAP methodology could reliably detect multiple islet autoantibodies in 1μL serum sample with strong performance.

## Discussion

Islet-cell immunoassays remain the technological fulcrum for efforts to diagnose and predict autoimmune diabetes. In this study, we developed and rigorously validated a multiplex islet autoantibody assay that can use as little as 1μL of serum sample. Through analysis of 7 cohorts comprised of 858 samples, we established the high performance of the assay in samples from relatives of T1D, as well as new-onset and established T1D. In particular, the high sensitivity of the assay was affirmed in the IASP 2018 study. The assay exhibited high specificity in analysis of samples containing excessive potentially interfering substances, such as hemoglobin, lipids and bilirubin, underscoring the resilience of the assay in measuring islet autoantibodies in challenging sample types. In addition, the assay also retained satisfactory specificity when testing healthy controls and diverse samples derived from type 2 diabetes, hyperglobulinemia and SLE patients. Notably, the assay also showed promise for risk screening by reliably identifying high-risk relatives with two or more autoantibodies.

It was noted that the control groups had differences in age, gender or races than the cases for few of the cohorts. Since the prevalence of islet autoantibodies was minimally dependent on these factors [23, 24], the difference should have marginal impact on the observed sensitivity and specificity. Indeed, the high sensitivities and specificities of the assay were observed in several well matched cohorts, such as the fully matched IASP 2018 study (cohort 2).

ADAP utilizes a unique mechanism for autoantibody detection. Firstly, ADAP detects all immunoglobulin isotypes, such as IgG, IgM and IgA [14, 15], whereas classic radioassay employs protein A as the precipitation agent that binds strongly with IgG and variably with IgA and IgM. Therefore, ADAP might identify autoantibody-positive patients that are not detected using current methods. Secondly, ADAP has been shown to be 100 to 10,000 times more analytically sensitive than conventional immunoassays [11, 12]. ADAP might therefore identify very low quantities of autoantibodies that would otherwise be missed by existing assays. Thirdly, ADAP demands at least two binding events between autoantibodies and antigens to trigger signal generation. Therefore cross-reactive autoantibodies such as those from SLE patient are likely rendered silent in the ADAP assay. Combined, these unique features of ADAP could explain its different comparative performance to radioassay for GAD, IA-2 and insulin autoantibodies.

For each autoantibody, the overall agreement between ADAP and radioassays were mostly above 90% across all cohorts. For those 10% of samples showing discrepant results, ADAP had a higher chance of identifying additional autoantibodies. For instance, among all T1D samples, ADAP identified an additional 25 GAD autoantibody positives, whereas radioassay detected 6 extra GAD positive samples. These additional ADAP positivity were likely a result of enhanced ADAP sensitivity since the sample signals were close to the cutoffs. Similarly, ADAP identified an additional 13 IA-2 and 15 insulin autoantibodies positive, whereas radioassay detected 4 and 9 negative in ADAP respectively. Consistent with these observations, ADAP’s ability to identify additional positivity may have contributed to its high performance in the IASP study.

Apart from ADAP, several research groups have developed and implemented non-radioactive assays for islet autoantibody measurement. Multiplex ELISA (also known as the three-screen ELISA) relies on a familiar ELISA workflow and instrumentation [8, 25]. It generates a single composite signal in the presence of GAD, IA-2 or ZnT8 autoantibodies. Confirmatory re-testing by radioassay is necessary to uncover insulin autoantibodies and to discriminate between individual autoantibody types and titers. Another example is the multiplex electrochemiluminescence assay (ECL). The ECL employs an acid dissociation step to process the serum sample, which is then incubated overnight (>16 hr) with probes before subsequent signal analysis for GAD, IA-2 and insulin autoantibodies [9–13]. Recently, this method was expanded to measure 7 autoantibodies in a single assay [13].

The multiplex ADAP assay is unique in that it leverages highly sensitive PCR amplification to further reduce the required sample volume for accurate analysis down to 1 μL of serum, eliminates the acid-dissociation step, and substantially reduces the assay incubation time down to 30 min. The sample-sparing (1 μL) feature is not only ideal for pediatric patients who may not be amenable to large volume blood draws, but also saves precious residual volume from samples for use in studies of genetic, transcriptomics, metabolic and cellular analyses. The non-radioactive and operationally simple workflow can significantly increase assay throughput. Lastly, ADAP relies on a commonly available real-time quantitative PCR (RT-qPCR) thermocycler for assay readout, thus eliminating the capital burden of acquiring specialty instrumentation.

The extensive validation of the ADAP assay presages a path to further expand its research and clinical utility. This includes large-scale longitudinal studies to more fully characterize the additional unique value of ADAP autoantibody signals in prediction of T1D risk and further comparative studies of ADAP to ELISA, ECL and other assay formats. Finally, incorporation of ZnT8 may also enhance the performance of the multiplex ADAP islet autoantibody assay [26]. It is noted however that ZnT8 autoantibody is rarely the lone autoantibody seen in isolation in subjects that progress to clinical diabetes [27, 28].

In summary, we report the development and validation of an ADAP assay as a sample-sparing and high-performing option for detecting islet autoantibodies. The method may also be applicable to other autoimmune diseases, as the DNA barcoding nature of ADAP made it possible to expand beyond currently 3-plex to encompass additional autoantigens. Importantly, the reduction of blood draw volume and the ability to reveal multiple autoantibodies with exceptional sensitivity and specificity from a single 1 μL sample may improve biomarker discovery, clinical trial enrollment and patient care in the pediatric population. If these favorable attributes are widely validated, the assay will become a valuable research and clinical tool for T1D and other immunological diseases.

## Materials and methods

### Materials

The full length GAD65 from RSR (#rGAD/FR/1) and insulin from Sigma Aldrich (#91077) were full-length recombinant proteins produced in yeast. The IA-2 antigen from RSR (#rIA2/FR/1) was a recombinant protein fragment from amino acid 604–979 produced in E. Coli. Polyclonal GAD antibodies were purchased from R&D systems (#AF2247). IA-2 antibodies were from Proteintech (#10584-1AP). Insulin antibodies were from Abcam (#14042). Platinum Taq polymerase (#10966026) and SYBR qPCR 2X master mix (#4385610) was purchased from Thermo Fisher. Hemoglobin (#H7379), bilirubin (#B4126) and intralipid (#I141) were purchased from Sigma Aldrich. Dithiothreitol (DTT #202090) and sulfo-SMCC (#22122) were purchased from Life Technologies. DNA ligase (#A8101) was purchased from Lucigen. Other reagents are detailed in the method sections as appropriate.

### Study population

Clinical samples used in this study were collected after written informed consent and with Institutional Review Board (IRB) approval at respective centers as appropriate. All of the clinical specimens in this study were received by Enable Biosciences as de-identified coded samples and determined by certified IRB professional (C.I.P.) from Western IRB to be exempt from the IRB review in 2017. A followed up IRB review (#20180015, Panel 05 from Western IRB) further approved Enable’s use of de-identified specimens for the study. Serum samples were obtained from multiple sources (S1 Table).

In cohort 0, serum from 200 healthy individuals and 120 established T1D patients were used to determine the assay cutoff thresholds. The healthy serum samples were provided by the Benaroya Research Institute (BRI) and BioIVT (median age of 33 years, 54% male, and 40% White). The established T1D serum samples were provided by the T1D Exchange (T1DX) from the “Metformin therapy for overweight adolescents with type 1 diabetes (NCT01881828)” clinical trial (median age of 15.4 years, 29% male, 78% White, a median disease duration of 7.1 years).

In cohort 1, the BRI provided archived serum from 30 established T1D patients (median age of 32 years, 57% male, 77% White, median duration of diseases:17.7 years) and 39 healthy control (median age of 33 years, 44% male, 72% White) to preliminarily evaluate the assay performance. This cohort was not blinded to those performing the assays.

In cohort 2, the coded IASP 2018 samples were received from the IASP Committee and the University of Florida [18] to compare performance with other laboratory in a blinded fashion. This cohort included 43 new-onset T1D samples within 14 days of diagnosis (median age of 14 years, 65% male, 86% white Caucasian, disease duration < 14 days), 7 multi-autoantibody positive first degree relatives of T1D (median age of 16 years, 43% male, 100% White) and 90 matched controls (median age of 20 years, 49% male, 77% White).

In cohort 3, the BRI provided coded serum samples from 20 established T1D (median age of 23 years, 35% male, 85% White, median disease duration of 15.2 years) and 30 type 2 diabetes (median age of 47 years, 33% male, 67% White) serum samples to evaluate ADAP performance in an independent blinded cohort.

In cohort 4, the Mayo Clinic provided blinded samples, including 20 serum from established T1D patients, 20 serum from normal controls, 6 from systemic lupus erythematosus (SLE) and 14 from hyperglobulinemia (HG). All these samples were remnant test samples from the Neuroimmunology Laboratory. In cohort 5, the Mayo Clinic further provided 80 coded remnant serum from established T1D patients.

In cohort 6, serum samples from Stanford University were collected at the Lucile Salter Packard Children Hospital. The samples included 32 established T1D patients (median age of 17 years, 56% male, median disease duration of 6 years), 18 new onset T1D (median age of 11.5 years, 44% male, median disease duration of 45 days), 39 first and second degree relatives of T1D (median age of 17 years, 44% male) and 50 normal controls (median age of 14 years, 46% male). The samples were analyzed in a blinded fashion.

### Radioassays

Radioassay testing for GAD, IA-2 and insulin autoantibodies of samples from the BRI and Stanford was performed by the Barbara Davis Center. The radioassay cutoffs for the Barbara Davis Center were 20 DK units/mL, 5 DK units/mL and 0.010 (index) for GAD, IA-2 and insulin autoantibodies, respectively (S4 Table). The radioassay cutoffs for the Neuroimmunology Laboratory at the Mayo Clinic were 0.02 (index) for all three autoantibodies. Cutoffs for radioassays were exclusively established by respective centers according to their clinical practice [29–31] and used directly in this study without further modifications. We did not re-determine the cutoffs of radioassays using samples from this study.

### Synthesis of islet cell antigen-DNA conjugates

For GAD65 and IA-2 conjugates, the proteins were buffer exchanged in reaction buffers (55 mM sodium phosphate, 150 mM sodium chloride, 20 mM EDTA, pH 7.2) to make 1 mg/mL solutions. A 1 μL solution of 8mM Sulfo-SMCC (Thermo Scientific) was added to 10 μL of the protein solution. The reaction mixture was incubated at room temperature for 2 h. Thiolated-DNA (Integrated DNA Technologies) was suspended in reaction buffers to 100 μM. A 3 μL solution of thiolated-DNA solution and 4 μL of 100 mM solution of DTT were mixed to reduce dimerized thiolated-DNA to monomer forms. The solution was then incubated at 37°C for 1 h. The excess sulfo-SMCC in protein mixtures and DTT in thiolated-DNA were removed by 7K MWCO Zeba spin column (Thermo Fisher). The thiolated-DNA and protein solutions were then pooled and incubated overnight at 4°C. The DNA-to-protein incubation ratio was 3-to-1 for all proteins. Finally, protein-DNA conjugates were purified by 30 kDa MWCO filter (Millipore). Conjugate concentrations were determined by BCA assay (Life Technologies). Conjugation efficiencies were analyzed by SDS-PAGE and silver staining as described previously. DNA-to-protein ratios of the conjugates were estimated by UV-VIS absorption and typically fell in the range of 2-to-1. Protein-DNA conjugates were stored at 4°C for short-term usage or aliquoted for long-term storage at -80°C. The insulin conjugate synthesis condition has been detailed elsewhere [11].

### ADAP type 1 diabetes assay

DNA barcoded islet antigens were used in the antibody detection by agglutination-PCR (ADAP) assay to measure GAD, IA-2 and insulin autoantibodies/antibodies in a single sample (Fig 1A). Briefly, 1 μL of serum sample was incubated with 2 μL conjugate mixtures (containing 1 femtomole of GAD, IA-2 and insulin-DNA conjugates) at 37°C for 30 min. Then, 116 μL of ligation mix (20 mM Tris, 50 mM KCl, 20 mM MgCl_2_, 20 mM DTT, 25 μM NAD, 0.025 U/μl ligase, 100 nM connector) was added and incubated at 30°C for 15 min. Then, 25 μL of ligated solution was mixed with PCR master mix that contained all primer pairs and amplified under standard thermocycling conditions (95°C for 10 min, 95°C for 15 sec, 56°C for 30 sec, 12 cycles). The pre-amplified products were then quantified by different primer pairs in a 96-well qPCR plate. SYBR green-based qPCR was performed on a Bio-Rad CFX96 real-time PCR detection system (95°C for 10 min, 95°C for 30 sec, 56°C for 1 min, 40 cycles). Instead of using the common cycle threshold (Ct) as readout, the ADAP assay readout ΔCt is defined as the Ct value of the blank control minus the Ct value of the actual sample (S7 Fig). The ΔCt for each DNA amplicon/primer pair was calculated accordingly. Therefore, ADAP achieved multiplex quantification of multiple antibody targets from a single sample.

The value of ΔCt is proportional to the initial amplicon concentrations in the PCR plate well, which in turn is proportional to the amount of target antibody present in the samples. The ΔCt offers significant reproducibility as the subtraction of blank control Ct and sample Ct cancels out any potential drift across runs.

The thresholds of positivity of the multiplex ADAP assay for GAD, IA-2 and insulin autoantibodies were determined by testing 200 healthy control (cohort 0). The GAD, IA-2 and insulin cutoffs were set at 99^th^ percentile of ΔCt from the normal controls, and were 2.39, 2.68 and 1.05 respectively. The same cutoffs were applied for all of the cohorts.

### Assay reproducibility

The intra-assay variation of ADAP was evaluated by measuring 5 replicates of samples on the same plate, whereas inter-assay variations were determined by testing 5 replicates of samples on 5 different days. The intra- and inter-assay variations were below 15% for autoantibody positive samples.

### Data analysis

PRISM (version 8.1.1) and XLSTAT software (version 2018.1) were used for data and statistical analysis. For the Pearson’s correlation analysis, radioassay signals were logarithmically transformed. The use of logarithm was necessary as ΔCt is a logarithmic parameter. (For instance, consider a sample of ΔCt value 2 and another sample of ΔCt of 4, their amplicon quantities differ by 4 fold (2^4^/2^2^) rather than 2 fold). The ROC analysis were performed to evaluate accuracy of ADAP and radioassays. For the concordance analysis, we used Cohen’s kappa statistics. Clinical sensitivity were calculated by dividing the number of T1D patients positive for at least one autoantibody by the total number of T1D patients, while the clinical specificity were calculated as the number of control patients negative for all autoantibodies divided by the total number of control patients. Two-tailed P values with an alpha of 0.05 were used as the cutoff for significance.

## Supporting information

S1 Fig Representative SDS-PAGE of antigen-DNA conjugates. Lane 1: Unconjugated GAD protein. Lane 2 and 3: GAD protein conjugated with DNA. Up shifts were observed due to increased molecular weight after chemical conjugation.
(TIF)

S2 Fig ADAP signals in cohort 0 of 120 T1D and 200 control serum samples. The signal distribution reached statistical significance between T1D (blue) and control (red) populations for all three autoantibodies (*p<0.05). The horizontal dash line represented cutoffs at the 99th percentile.
(TIF)

S3 Fig ADAP was tolerant for varying concentration of lipids and bilirubin. **(A)** Varying concentrations of lipids were spiked into T1D and control serum. The normal lipid level should be lower than 200 mg/dL No interference is observed up to 3000 mg/dL of hemoglobin. **(B)** Varying concentrations of bilirubin were spiked into T1D and control serum. The normal bilirubin level should be lower than 1.2 mg/dL. No interference is observed up to 6.6 mg/dL of bilirubin.
(TIF)

S4 Fig Analysis of 80 T1D serum samples from Mayo Clinic. The x-axis displays radioassay signals in logarithm scales. The y-axis shows ADAP signal in ΔCt. The horizontal and the vertical dash lines denote ADAP and radioassay cutoff thresholds respectively. ADAP identified additional positive samples.
(TIF)

S5 Fig Analysis of at-risk, new onset and established T1D serum samples from Stanford School of Medicine. The x-axis displays radioassay signals in logarithm scales. The y-axis shows ADAP signal in ΔCt. The horizontal and the vertical dash lines denote ADAP and radioassay cutoff thresholds respectively. Data from each sample group is color coded (blue circle for established T1D, purple diamond for new onset T1D, green triangle for at-risk relatives of T1D, red square for control).
(TIF)

S6 Fig ROC plots of pooled raw data from cohort 1, 3 and 6. The ROC curves of ADAP showed AUC of 0.91 (95%CI: 0.87–0.95), 0.82 (95%CI: 0.76–0.88) and 0.95 (95%CI: 0.92–0.98) for GAD, IA-2 and insulin antibodies/autoantibodies respectively. The samples were also analyzed by radioassay and showed corresponding AUC of 0.91 (95%CI: 0.86–0.95), 0.83 (95%CI: 0.77–0.89) and 0.96 (95%CI: 0.94–0.99). The AUC between ADAP and radioassay was not statistically distinguishable. Noted that cohort 4 and cohort 5 were not included since their radioassays were performed by Mayo Clinic, whose assay performance had not been correlated with those at Barbara Davis Center.
(TIF)

S7 Fig Representative real-time quantitative PCR (qPCR) curves for ADAP experiments. In a standard qPCR experiment, fluorescent values (arbitrary unit au, y-axis) would gradually increase as PCR cycling went on (Cycle number, x-axis). For instance, here we illustrated representative qPCR curves for T1D serum samples using GAD-DNA conjugates. The Ct value of qPCR was defined as the cycle number where fluorescent readout of the sample equaled a defined threshold fluorescent value (black horizontal dash line). The Ct value of T1D positive serum was 19.86 (Ct1, blue vertical dash line), whereas Ct value of healthy samples was 28.80 (Ct2, pink vertical dash line) and buffer only blank were 29.09 (Ct3, green vertical dash line). The ΔCt of an ADAP experiment was defined as the Ct value difference between a sample and a blank control. Therefore, the ΔCt for T1D serum will be 9.23 (29.09–19.86), and ΔCt for healthy serum will be 0.29 (29.09–28.80). A larger ΔCt indicated that the sample contained higher amount of PCR amplicons, which then reflected the presence of higher amount of antibodies/autoantibodies.
(TIF)

S1 Table Summary of patient cohorts involved in this study. Cohort 0 was the assay training cohort used to establish the assay cutoff thresholds. Cohort 1 to cohort 6 were assay validation cohorts. Notably, cohort 2 was from the Islet Autoantibody Standardization Program (IASP). Sensitivity and specificity of all participating methods in IASP were made public available by the committee but not individual data points, and thus IASP cohort is not included in those cross-cohort analysis requiring individual sample signals.
(DOCX)

S2 Table Summary of clinical performance of ADAP assay across cohorts. Sensitivity is defined as the percentage of T1D samples with one or more autoantibodies. Specificity is defined as the percentage of control or non-T1D samples without any autoantibodies. Noted that in cohort 6, 39 relatives of T1D were excluded from the analysis, as they have yet developed any clinical symptoms for definitive diagnosis.
(DOCX)

S3 Table Summary of correlation, concordance and agreements between ADAP and radioassay across cohorts. As noted above, the radioassay signals for individuals samples from participating laboratory are not publicly available. Thus, the correlation, concordance and agreement was not reported for that cohort.
(DOCX)

S4 Table Cutoffs for various assays involved in the study. The cutoffs of radioassays performed by the Barbara Davis Center and Mayo Clinic were established by each center according to their routine clinical practice and the procedures have been reported previously [8–10]. We did not re-determine the cutoffs of radioassays using any samples from this study.
(DOCX)
