# Supplementary figures and images for: Sensitive detection of multiple islet autoantibodies in type 1 diabetes using small sample volumes by agglutination-PCR
Source: PLoS One. 2020 Nov 13;15(11):e0242049. doi: 10.1371/journal.pone.0242049 (PMC7665791; doi:10.1371/journal.pone.0242049)

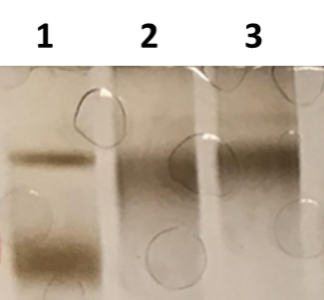

Supplement: S1 Fig — Lane 1: Unconjugated GAD protein. Lane 2 and 3: GAD protein conjugated with DNA. Up shifts were observed due to increased molecular weight after chemical conjugation. (TIF) [file pone.0242049.s001.tif]

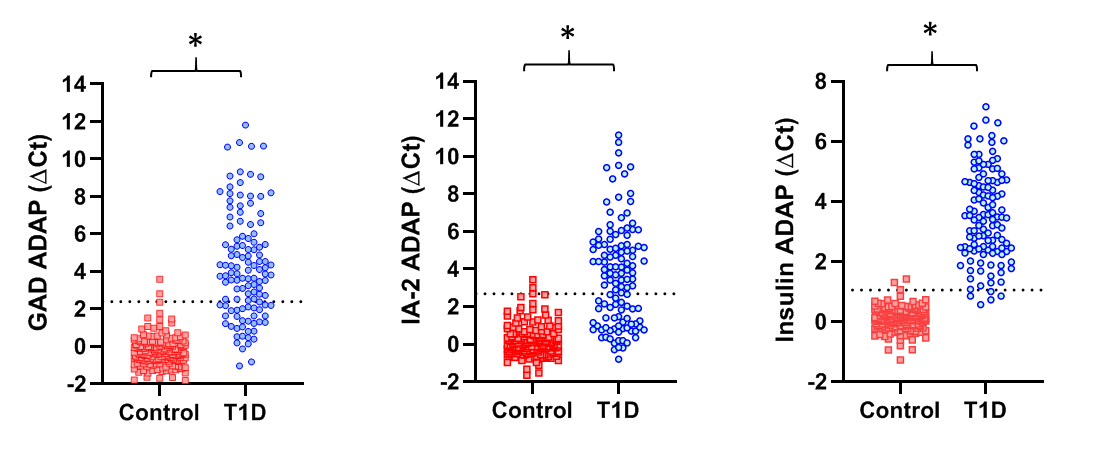

Supplement: S2 Fig — The signal distribution reached statistical significance between T1D (blue) and control (red) populations for all three autoantibodies (*p<0.05). The horizontal dash line represented cutoffs at the 99th percentile. (TIF) [file pone.0242049.s002.tif]

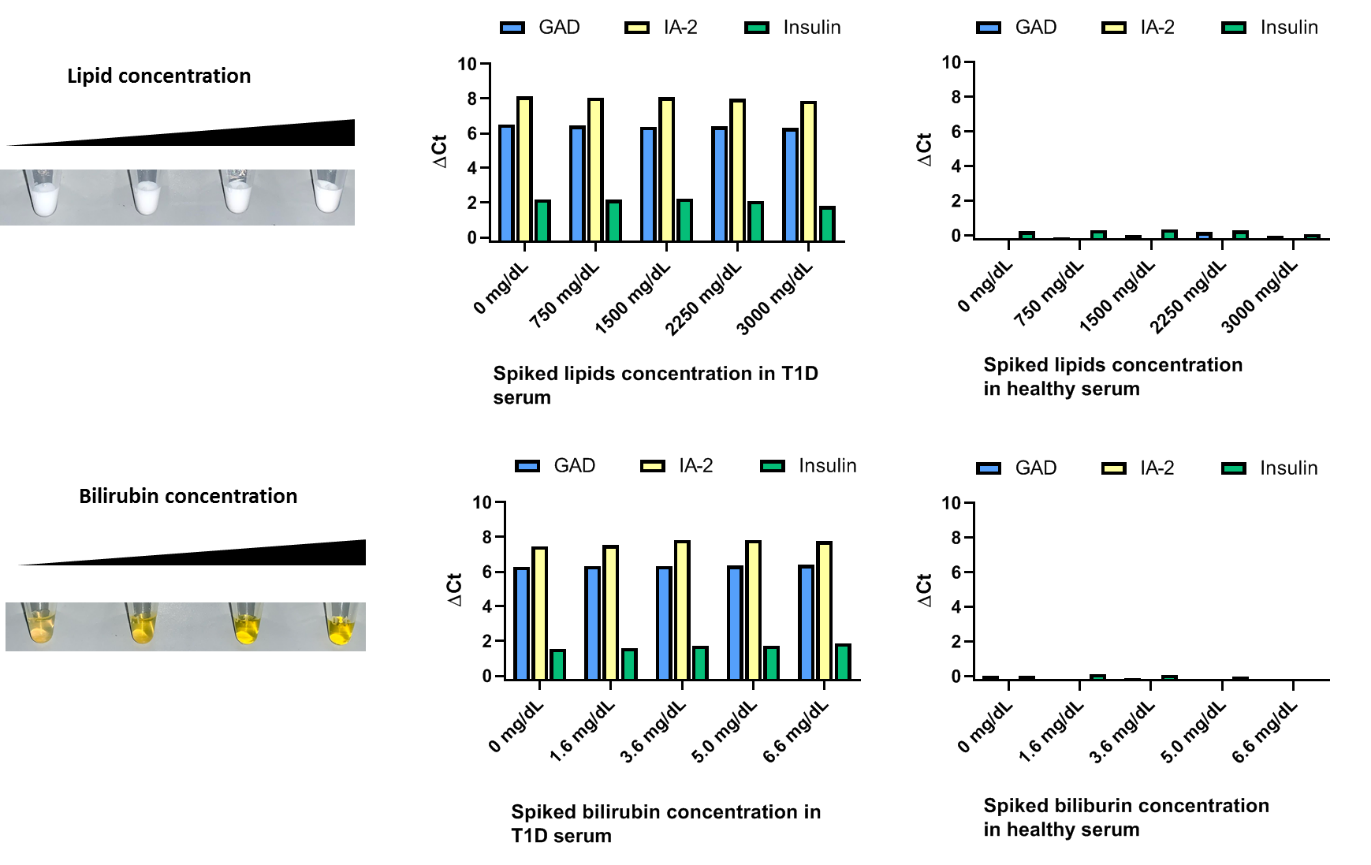

Supplement: S3 Fig — (A) Varying concentrations of lipids were spiked into T1D and control serum. The normal lipid level should be lower than 200 mg/dL No interference is observed up to 3000 mg/dL of hemoglobin. (B) Varying concentrations of bilirubin were spiked into T1D and control serum. The normal bilirubin level should be lower than 1.2 mg/dL. No interference is observed up to 6.6 mg/dL of bilirubin. (TIF) [file pone.0242049.s003.tif]

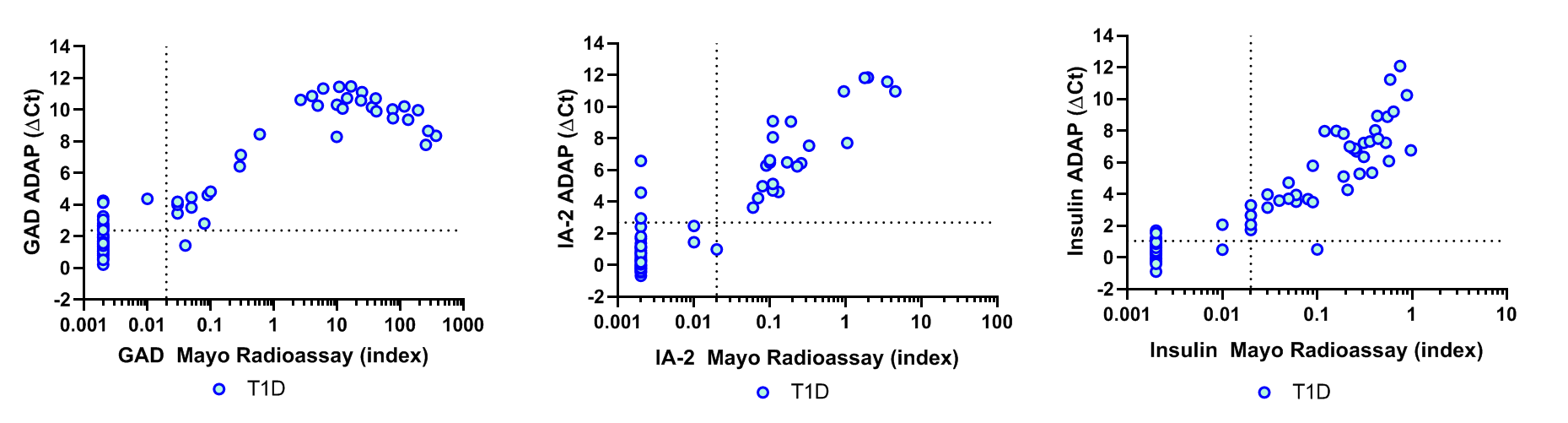

Supplement: S4 Fig — The x-axis displays radioassay signals in logarithm scales. The y-axis shows ADAP signal in ΔCt. The horizontal and the vertical dash lines denote ADAP and radioassay cutoff thresholds respectively. ADAP identified additional positive samples. (TIF) [file pone.0242049.s004.tif]

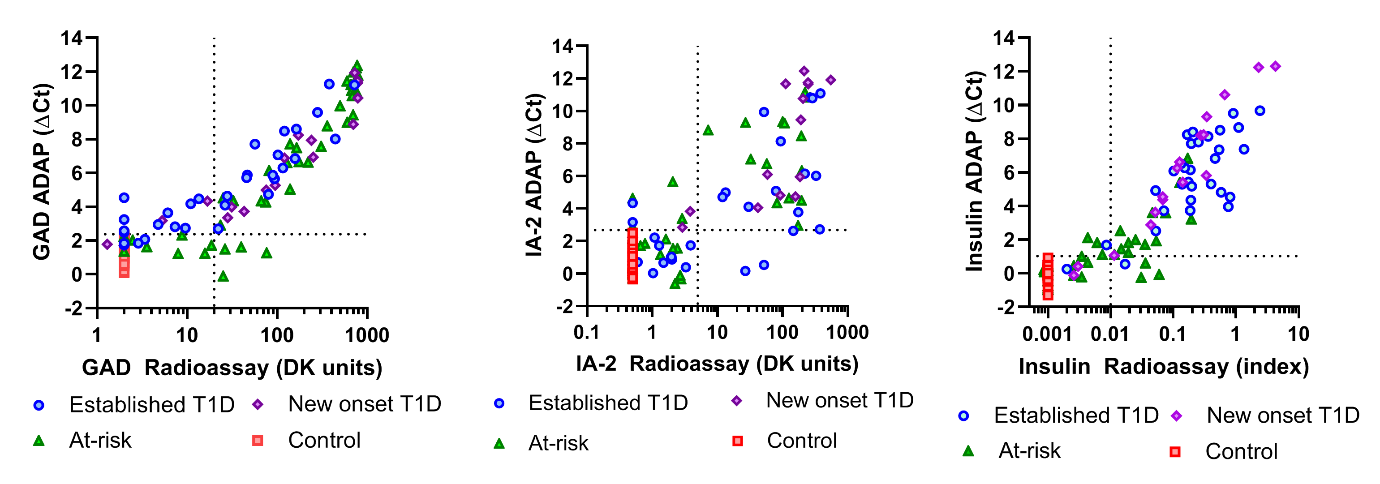

Supplement: S5 Fig — The x-axis displays radioassay signals in logarithm scales. The y-axis shows ADAP signal in ΔCt. The horizontal and the vertical dash lines denote ADAP and radioassay cutoff thresholds respectively. Data from each sample group is color coded (blue circle for established T1D, purple diamond for new onset T1D, green triangle for at-risk relatives of T1D, red square for control). (TIF) [file pone.0242049.s005.tif]

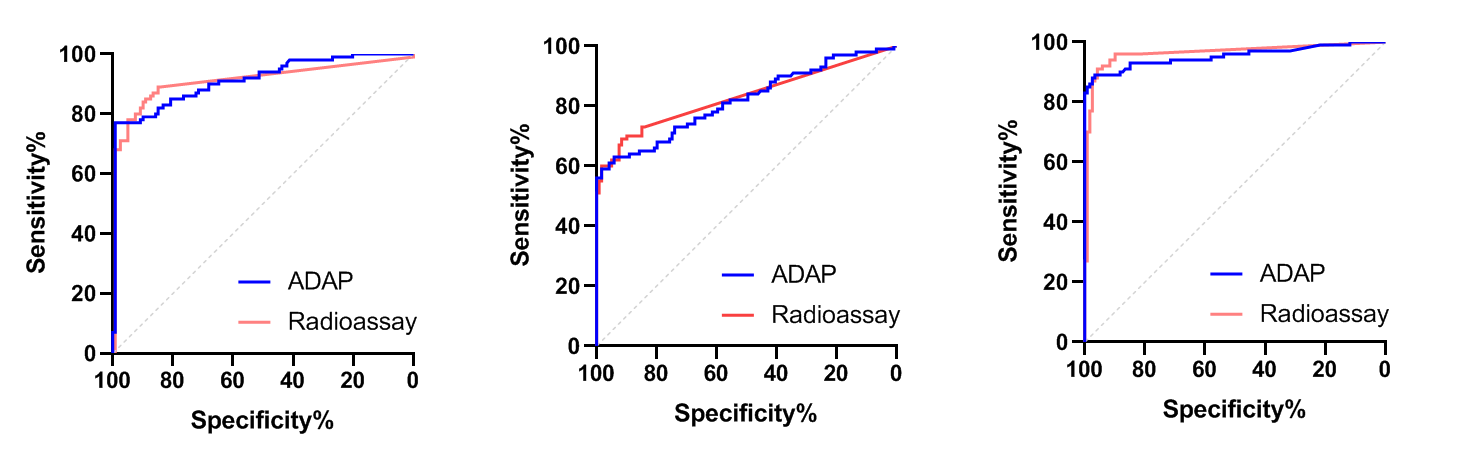

Supplement: S6 Fig — The ROC curves of ADAP showed AUC of 0.91 (95%CI: 0.87–0.95), 0.82 (95%CI: 0.76–0.88) and 0.95 (95%CI: 0.92–0.98) for GAD, IA-2 and insulin antibodies/autoantibodies respectively. The samples were also analyzed by radioassay and showed corresponding AUC of 0.91 (95%CI: 0.86–0.95), 0.83 (95%CI: 0.77–0.89) and 0.96 (95%CI: 0.94–0.99). The AUC between ADAP and radioassay was not statistically distinguishable. Noted that cohort 4 and cohort 5 were not included since their radioassays were performed by Mayo Clinic, whose assay performance had not been correlated with those at Barbara Davis Center. (TIF) [file pone.0242049.s006.tif]

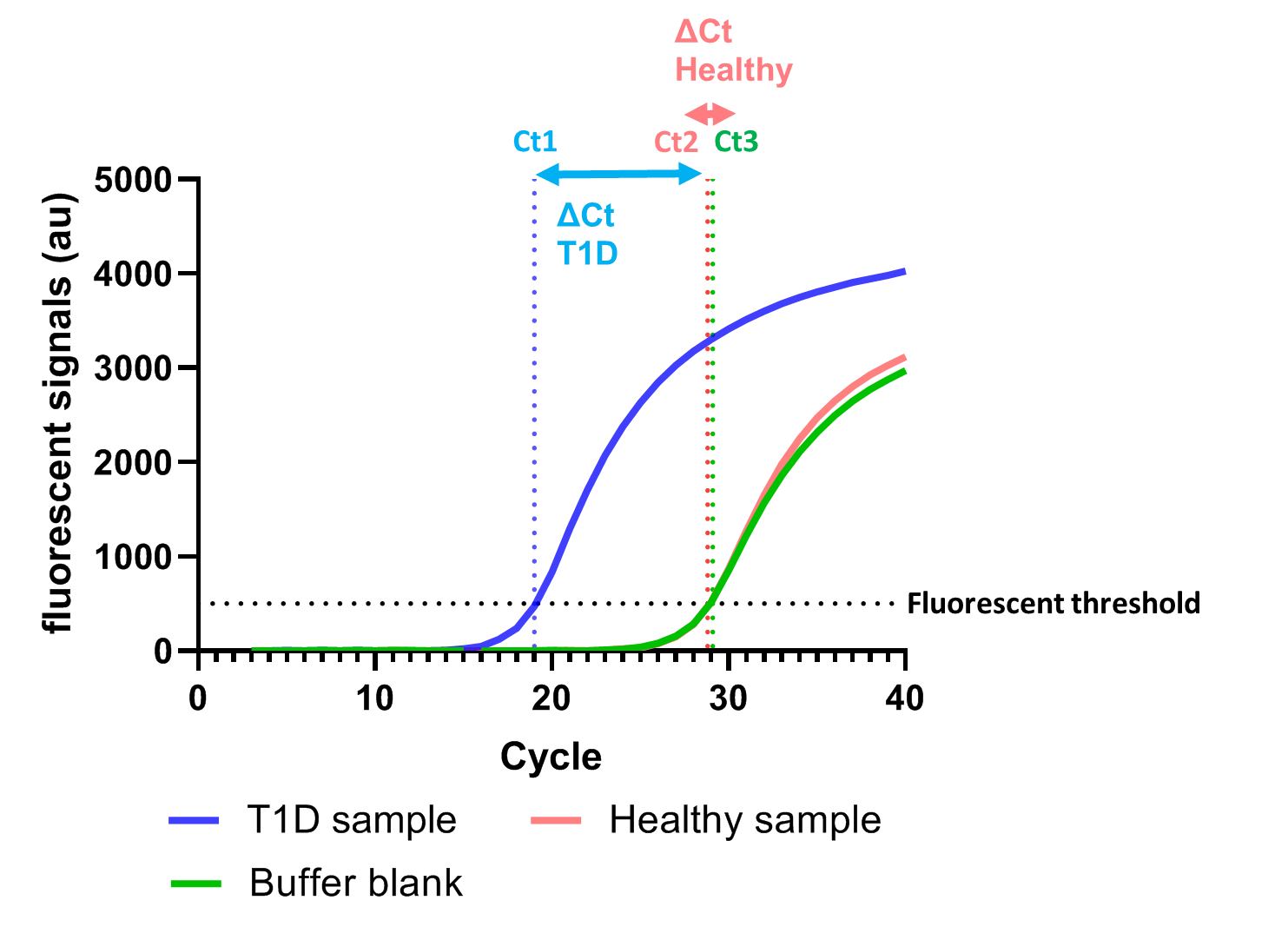

Supplement: S7 Fig — In a standard qPCR experiment, fluorescent values (arbitrary unit au, y-axis) would gradually increase as PCR cycling went on (Cycle number, x-axis). For instance, here we illustrated representative qPCR curves for T1D serum samples using GAD-DNA conjugates. The Ct value of qPCR was defined as the cycle number where fluorescent readout of the sample equaled a defined threshold fluorescent value (black horizontal dash line). The Ct value of T1D positive serum was 19.86 (Ct1, blue vertical dash line), whereas Ct value of healthy samples was 28.80 (Ct2, pink vertical dash line) and buffer only blank were 29.09 (Ct3, green vertical dash line). The ΔCt of an ADAP experiment was defined as the Ct value difference between a sample and a blank control. Therefore, the ΔCt for T1D serum will be 9.23 (29.09–19.86), and ΔCt for healthy serum will be 0.29 (29.09–28.80). A larger ΔCt indicated that the sample contained higher amount of PCR amplicons, which then reflected the presence of higher amount of antibodies/autoantibodies. (TIF) [file pone.0242049.s007.tif]
