# Supplementary material for: Sensitive detection of multiple islet autoantibodies in type 1 diabetes using small sample volumes by agglutination-PCR
Source: PLoS One. 2020 Nov 13;15(11):e0242049. doi: 10.1371/journal.pone.0242049 (PMC7665791; doi:10.1371/journal.pone.0242049)
Supplement: S1 Table — Cohort 0 was the assay training cohort used to establish the assay cutoff thresholds. Cohort 1 to cohort 6 were assay validation cohorts. Notably, cohort 2 was from the Islet Autoantibody Standardization Program (IASP). Sensitivity and specificity of all participating methods in IASP were made public available by the committee but not individual data points, and thus IASP cohort is not included in those cross-cohort analysis requiring individual sample signals. (DOCX) [file pone.0242049.s008.docx]

|  | Cohort 6 | Cohort 5 | Cohort 4 | Cohort 3 | Cohort 2 | Cohort 1 | Cohort 0 | Cohort ID |  |
| --- | --- | --- | --- | --- | --- | --- | --- | --- | --- |
|  | Stanford | Mayo | Mayo | BRI | IASP 2018 | BRI | BioIVT, BRI, T1DX |  | Source |
| Total 858 | 139 | 80 | 60 | 50 | 140 | 69 | 320 |  | Number of samples |
| Total 302 | 32 | 80 | 20 | 20 |  | 30 | 120 |  | Established T1D |
| Total 61 | 18 | ----- | ----- | ----- | 43 | ----- | 0 |  | New onset T1D |
| Total 46 | 39 | ----- | ----- | ----- | 7 | ----- | 0 |  | At-risk T1D |
| Total 449 | 50 | NA | 20 Controls, 6 SLE, 14 HG | 30 (T2D) | 90 | 39 | 200 |  | Controls |
|  | Yes | Yes | Yes | Yes | Yes | No | No |  | Blinded |
|  | Barbara Davis | Mayo | Mayo | Barbara Davis | N/A | Barbara Davis BRI | ---- |  | RBA sites |
