# Supplementary material for: Sensitive detection of multiple islet autoantibodies in type 1 diabetes using small sample volumes by agglutination-PCR
Source: PLoS One. 2020 Nov 13;15(11):e0242049. doi: 10.1371/journal.pone.0242049 (PMC7665791; doi:10.1371/journal.pone.0242049)
Supplement: S2 Table — Sensitivity is defined as the percentage of T1D samples with one or more autoantibodies. Specificity is defined as the percentage of control or non-T1D samples without any autoantibodies. Noted that in cohort 6, 39 relatives of T1D were excluded from the analysis, as they have yet developed any clinical symptoms for definitive diagnosis. (DOCX) [file pone.0242049.s009.docx]

| Cohort ID | Source | Number of samples | T1D  samples | Control samples | Blinded | Sensitivity | Specificity |
| --- | --- | --- | --- | --- | --- | --- | --- |
| Cohort1 | BRI | 69 | 30 | 39 | No | 93% | 100% |
| Cohort2 | IASP2018 | 140 | 50 | 90 | Yes | 95% | 99% |
| Cohort3 | BRI | 50 | 20 | 30 | Yes | 100% | 97% |
| Cohort4 | Mayo | 60 | 20 | 40 | Yes | 100% | 93% |
| Cohort5 | Mayo | 80 | 80 | NA | Yes | NA | NA |
| Cohort6 | Stanford | 89* | 50* | 50 | Yes | 93% | 100% |
|  |  |  |  |  | Average | 96% | 97% |
|  |  |  |  |  |  |  |  |
