# Supplementary material for: Sensitive detection of multiple islet autoantibodies in type 1 diabetes using small sample volumes by agglutination-PCR
Source: PLoS One. 2020 Nov 13;15(11):e0242049. doi: 10.1371/journal.pone.0242049 (PMC7665791; doi:10.1371/journal.pone.0242049)
Supplement: S3 Table — As noted above, the radioassay signals for individuals samples from participating laboratory are not publicly available. Thus, the correlation, concordance and agreement was not reported for that cohort. (DOCX) [file pone.0242049.s010.docx]

|  | Cohort 6 | Cohort 5 | Cohort 4 | Cohort 3 | Cohort 2 | Cohort 1 | Cohort ID |  |
| --- | --- | --- | --- | --- | --- | --- | --- | --- |
|  | Stanford | Mayo | Mayo | BRI | IASP 2018 | BRI |  | Source |
| Total 538 | 139 | 80 | 60 | 50 | 140 | 69 |  | Number of samples |
| Total 289 | 89 | 80 | 20 | 20 | 50 | 30 |  | Relatives of T1D and TTD |
| Total 249 | 50 | ---- | 40 (C, SLE, HG) | 30 (T2D) | 90 | 39 |  | Controls |
|  | Yes | Yes | Yes | Yes | Yes | No |  | Blinded |
| Average | Barbara Davis | Mayo | Mayo | Barbara Davis | Multiple labs | Barbara Davis |  | RBA sites |
| 0.88 | 0.85 | 0.93 | 0.94 | 0.86 | NA | 0.82 | GAD | Pearson’s correlation coefficients R |
| 0.79 | 0.77 | 0.93 | 0.97 | 0.71 | NA | 0.69 | IA2 |  |
| 0.88 | 0.88 | 0.93 | 0.94 | 0.79 | NA | 0.88 | INS |  |
| 0.77 | 0.88 | 0.75 | 0.69 | 0.80 | NA | 0.89 | GAD | Concordance coefficient  κ |
| 0.81 | 0.77 | 0.83 | 0.91 | 0.73 | NA | 0.92 | IA2 |  |
| 0.84 | 0.79 | 0.82 | 0.78 | 0.87 | NA | 0.86 | INS |  |
| 91% | 88% | 88% | 92% | 92% | NA | 96% | GAD | Overall agreement |
| 95% | 91% | 96% | 98% | 92% | NA | 97% | IA2 |  |
| 94% | 93% | 91% | 93% | 92% | NA | 93% | INS |  |
