# Supplementary material for: Sensitive detection of multiple islet autoantibodies in type 1 diabetes using small sample volumes by agglutination-PCR
Source: PLoS One. 2020 Nov 13;15(11):e0242049. doi: 10.1371/journal.pone.0242049 (PMC7665791; doi:10.1371/journal.pone.0242049)
Supplement: S4 Table — The cutoffs of radioassays performed by the Barbara Davis Center and Mayo Clinic were established by each center according to their routine clinical practice and the procedures have been reported previously [8–10]. We did not re-determine the cutoffs of radioassays using any samples from this study. (DOCX) [file pone.0242049.s011.docx]

|  | Barbara Davis Center Radioassays | Mayo Clinic  Radioassays | ADAP |
| --- | --- | --- | --- |
| GAD | 20 | 0.02 | 2.39 |
| IA-2 | 5 | 0.02 | 2.68 |
| INS | 0.01 | 0.02 | 1.05 |
